# Gallbladder Volvulus Masquerading as Acute Cholecystitis: A Case Report

**DOI:** 10.7759/cureus.48529

**Published:** 2023-11-08

**Authors:** Julie Chugh, Bayli Davis, Jeannette Sandoval, Basem Soliman

**Affiliations:** 1 Department of Surgery, Texas Tech University Health Sciences Center, Amarillo, USA

**Keywords:** cholecystitis, cholecystectomy, gallbladder disease, gallbladder torsion, gallbladder volvulus

## Abstract

Gallbladder volvulus is a rare condition that disproportionately affects elderly women. It occurs in patients with aberrant anatomy that results in a hypermobile gallbladder free to twist on the mesentery, leading to ischemia and necrosis. Due to its close resemblance to cholecystitis, it is difficult to diagnose preoperatively, thus most cases are diagnosed intraoperatively. In our case, a 90-year-old female was transferred to the hospital from an outside facility after being diagnosed with acute cholecystitis. A robotic-assisted laparoscopic approach was used to gain entry into the abdomen. Upon entry, the gallbladder was gangrenous, detached from the liver bed, and twisted on the cystic duct. Despite the presence of severe inflammatory changes and adhesions, the gallbladder was resected without complications, and the patient was discharged on postoperative day five.

## Introduction

Gallbladder volvulus is a known but rare condition, with about 500 cases reported in the literature [1]. It occurs when the gallbladder twists around the mesentery and typically presents as an acute abdomen in the elderly population. It is rarely diagnosed preoperatively due to inconclusive radiologic findings, and patients are often presumed to have acute cholecystitis until the diagnosis is made intraoperatively. Due to the potential for gallbladder ischemia and necrosis arising from entrapment of the cystic artery, emergent surgical intervention is warranted. Surgical intervention with laparoscopic cholecystectomy is the mainstay of treatment for gallbladder volvulus [2]. Medical management alone with antibiotics, percutaneous gallbladder drainage, and therapeutic endoscopy with endoscopic retrograde cholangiopancreatography (ERCP) have demonstrated limited effectiveness for the treatment of gallbladder volvulus in the literature [3,4].

## Case presentation

Our patient was a 90-year-old female who presented to her primary care physician (PCP) with complaints of abdominal pain for two days. The abdominal pain was described by the patient as sharp in quality, moderate in severity, and constant throughout the day. Related symptoms included nausea, diarrhea, and decreased appetite. The patient denied vomiting, fever, dysuria, and chest pain. No aggravating or alleviating factors were reported. The patient’s past medical history was significant for essential hypertension, irritable bowel syndrome with diarrhea, chronic obstructive pulmonary disease, and dementia. Past surgical history was significant for a left nephrectomy, tonsillectomy, and partial mastectomy. Physical exam at this time was significant for a distended abdomen, hypoactive bowel sounds, and tenderness to palpation in the right upper quadrant with mild guarding, per documentation.

**Figure 1:**
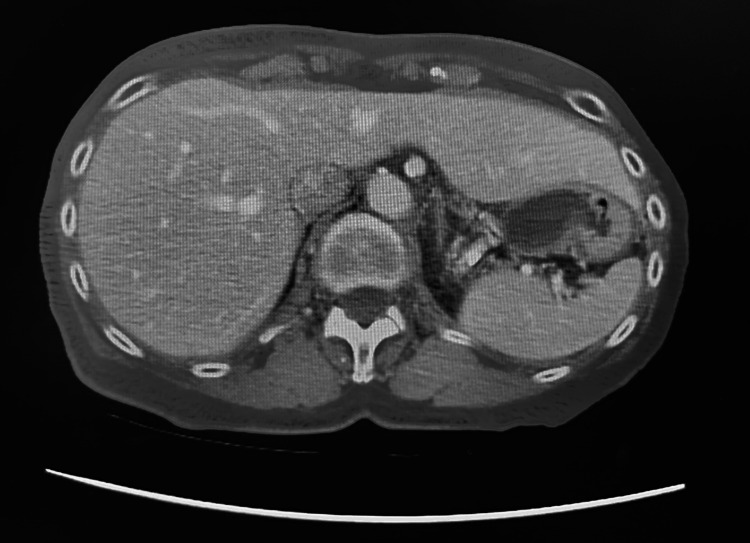
CT showing empty gallbladder fossa

**Figure 2:**
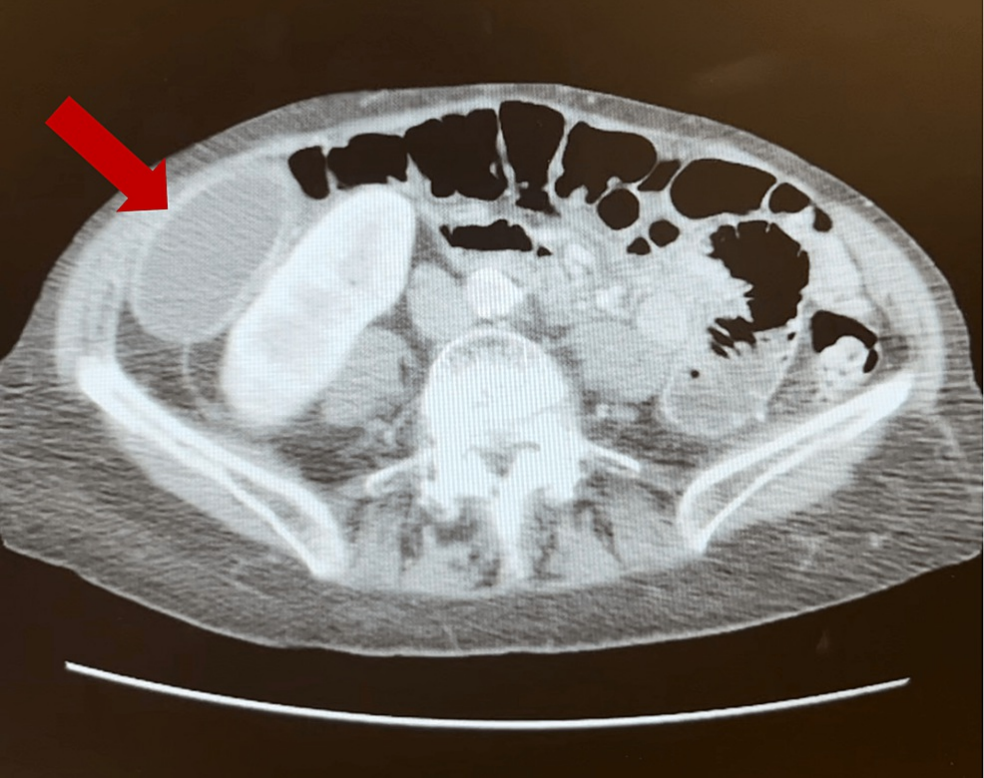
CT showing gallbladder in the right paracolic gutter/lateral to the right kidney

**Figure 3:**
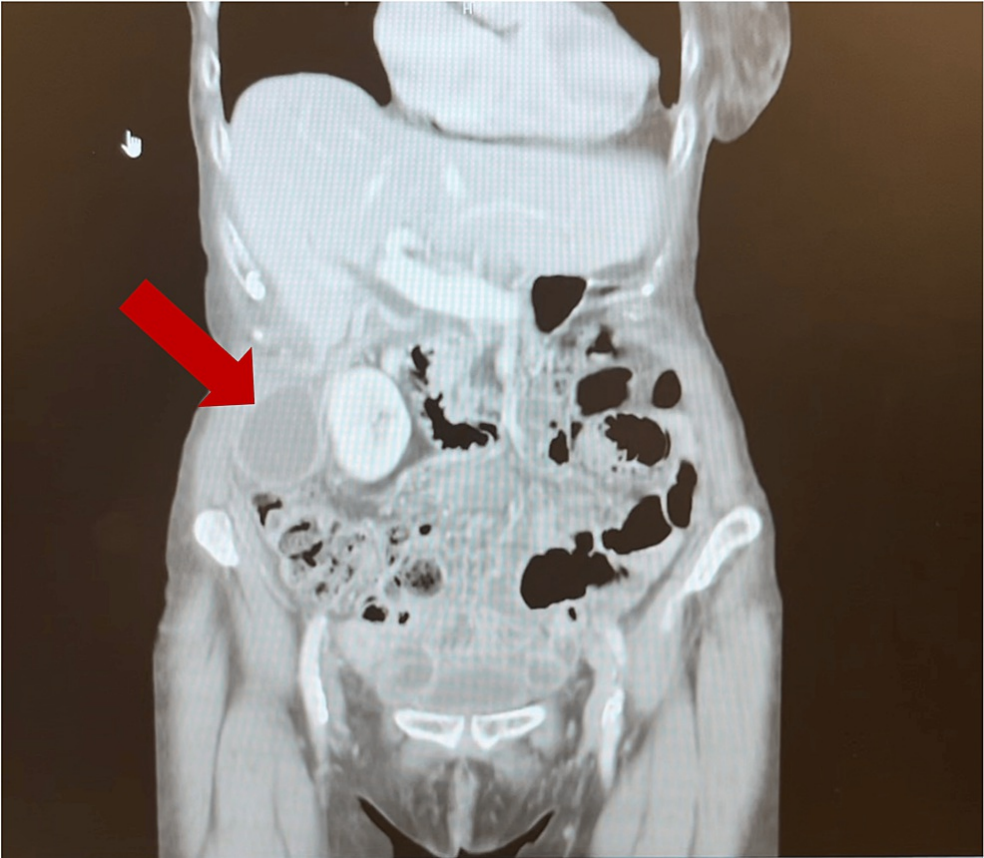
Alternative view of CT showing the gallbladder in the paracolic gutter/lateral to the right kidney

Laboratory studies conducted through the patient’s PCP included a complete blood count (CBC), comprehensive metabolic panel (CMP), lipase, creatinine kinase (CK), C-reactive protein (CRP), and troponin. Laboratory findings were significant for leukocytosis with a left shift and elevated CRP (Table 1). Computed tomography (CT) showed a distended gallbladder in the pelvis with wall thickening (Figures 1-3). Per the documentation, an abdominal ultrasound was performed and further demonstrated a dilated gallbladder with a small amount of sludge and possible pericholecystic fluid, though only the radiologic report was able to be obtained. Both imaging studies noted the possibility of acute cholecystitis as the diagnosis. At this point, the patient was referred to a general surgeon for evaluation and treatment options.

**Table 1:** Significant lab values collected by the patient’s PCP PCP: primary care provider

	Value	Normal Range
White Blood Cell (WBC)	11.5 x10E3/µL	3.9-10.0 x10E3/µL
Neutrophils	86.9%	
CRP	1.76 mg/dL	0.00-0.50 mg/dL

Two days after her visit to her PCP and four days before her appointment with the general surgeon, the patient presented to her local emergency department with persistent complaints of right upper quadrant pain and nausea. The physical exam performed at this time was consistent with what was documented by her PCP. Laboratory studies at this time were significant for worsening WBC and CRP levels (Table 2). Due to the patient’s worsening leukocytosis, elevated CRP, and advanced age, the decision was made to transfer the patient to a hospital in Amarillo, Texas, for a higher level of care. General surgery was consulted at the time of the patient’s arrival. Upon review of the patient’s history and previous workup, including laboratory and imaging results, a diagnosis of cholecystitis was made. The patient was scheduled for robot-assisted laparoscopic cholecystectomy that day.

**Table 2:** Significant lab values collected by the emergency department prior to the patient’s transfer to Amarillo, TX

	Value	Normal Range
WBC	13.2 x10E3/µL	4.0-11.0 x10E3/µL
Neutrophils	87%	
CRP	10.5 mg/dL	0.0-0.8 mg/dL

**Figure 4:**
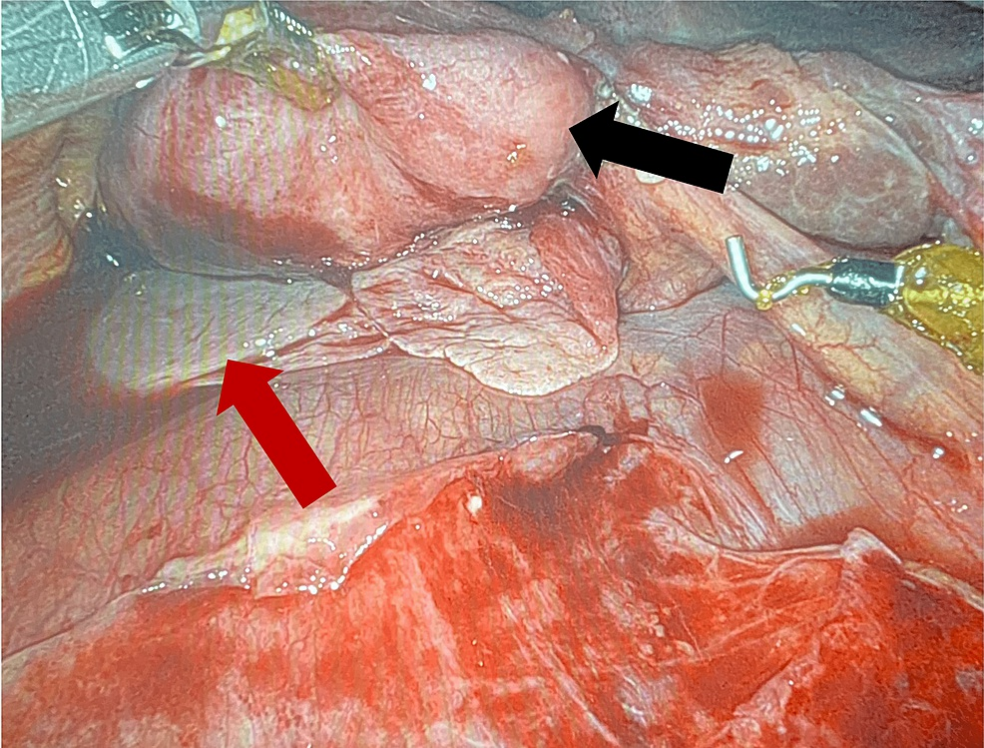
Intraoperative image demonstrating the inferior portion of the liver (red arrow) and the gallbladder nearly detached from the liver bed (black arrow)

**Figure 5:**
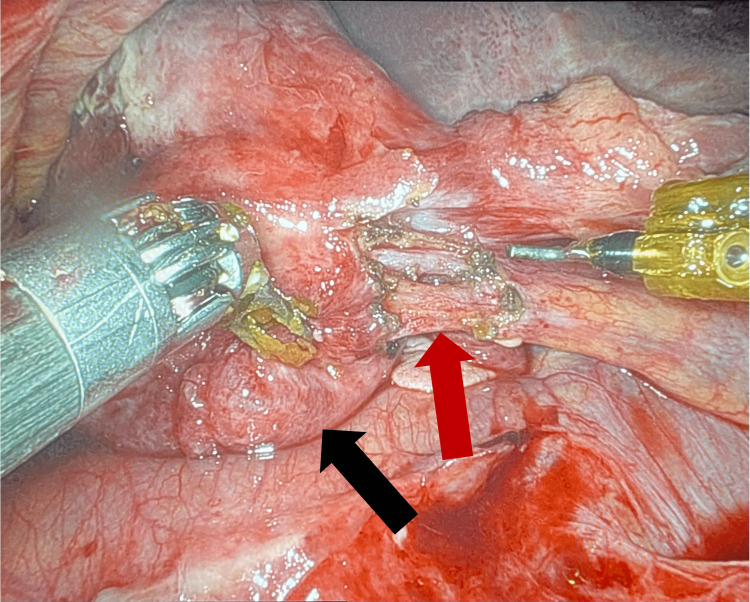
Intraoperative image demonstrating the gallbladder (black arrow) and the cystic duct (red arrow) that was previously twisted

Upon entrance into the abdominal cavity, severe inflammatory changes were noted around the gallbladder. Duodenal and transverse colonic adhesions in the right upper quadrant obscured the gallbladder. Once the gallbladder was reached, it was noted that it was gangrenous, nearly detached from the bed of the liver, and twisted on the cystic duct (Figures 4, 5). The gallbladder and its associated vascular and biliary attachments were dissected from surrounding structures via electrocautery hook dissection with the aid of indocyanine green (ICG)-enhanced cholangiography. There were no intraoperative complications, such as bleeding or bile leak, and the patient was transferred to the recovery room in stable condition. The patient’s recovery course was complicated by altered mental status and confusion, secondary to hospital-induced delirium, which resolved by the time of discharge. She was discharged on postoperative day five to a skilled nursing facility with a follow-up appointment scheduled in two weeks. The patient did not return for her follow-up appointment.

## Discussion

Gallbladder volvulus is a rare condition that disproportionately affects elderly patients [5]. Among this population, the ratio of females to males affected is four to one, and the median age of patients with this condition is 77 years old [6,7]. The primary etiology of gallbladder volvulus is largely unknown, but it is hypothesized that it occurs in predisposed individuals with one of four anatomical variants. The first variant is a complete absence of the gallbladder mesentery caused by abnormal migration of the pars cystica in the early embryonic phase of development. In this variant, the gallbladder is suspended only by the cystic duct and artery. The second variant occurs when the normally developed mesentery elongates and loses elasticity, allowing the gallbladder to effectively hang from the liver. Combined with liver atrophy, this variant may cause visceroptosis of the gallbladder and thus increased mobility. This variant is considered a normal part of aging and may contribute to the condition’s predilection for elderly patients. The third variant occurs when the gallbladder is detached from the liver bed. The fourth variant occurs when the gallbladder is fixed to a mobile hepatic lobe that is not adequately fixed via the coronary and triangular ligaments [5,6,8,9].

In patients with anatomical variants predisposing them to gallbladder volvulus, a precipitating event typically occurs that causes the torsion of the gallbladder [8]. Factors that may precipitate torsion include atherosclerosis of the cystic artery, kyphoscoliosis, gastric, duodenal, or colonic peristalsis, abdominal trauma, adhesions, rapid weight loss, constipation, iatrogenic manipulation, and more [7,8,10]. All four anatomic variants mentioned result in an abnormally mobile, “floating” gallbladder predisposed to torsion. In our patient, it was noted intraoperatively that the gallbladder was almost entirely detached from the liver. This finding, in combination with her advanced age, suggests that she likely suffered from the second or third anatomical variant due to age-related elongation and loss of elasticity of the gallbladder mesentery. The precipitating event that caused the volvulus was not determined.

Clinical and laboratory findings in patients with gallbladder volvulus are nonspecific and often mimic those found in patients suffering from acute cholecystitis. These symptoms include right upper quadrant pain, nausea and vomiting, palpable mass, and hyperbilirubinemia. It has been noted that the additional presence of fever, leukocytosis, jaundice, and poor response to antibiotics may be more indicative of gallbladder volvulus rather than acute cholecystitis [8,11]. Our patient’s demographics, clinical symptoms, and laboratory and intraoperative findings were suggestive of gallbladder volvulus, though acute cholecystitis could not be ruled out.

Abdominal ultrasound, the gold-standard imaging for patients who present with signs and symptoms of acute cholecystitis, is rarely able to differentiate acute cholecystitis from gallbladder volvulus, as it typically shows gallbladder wall thickening with pericholecystic fluid [7,12]. Some studies suggest, however, that if clinical suspicion for gallbladder volvulus is high, radiologists may perform more detailed ultrasounds through which one may find a largely distended gallbladder (larger than what would be seen in acute cholecystitis), a freely mobile gallbladder, a markedly thickened and multilayered gallbladder wall, an inferiorly displaced and horizontally-orientated gallbladder, or a conical structure connecting the gallbladder to the liver consistent with a twisted pedicle. It is important to note, however, that these findings are exceptional [7,11-13]. Doppler ultrasound showing decreased blood flow through the pedicle or an absence of flow to the gallbladder wall can also support the diagnosis of gallbladder volvulus rather than acute cholecystitis [13,14]. CT scan findings may also suggest a diagnosis of gallbladder volvulus. These include a marked enlargement of the gallbladder (larger than would be expected in other conditions that cause gallbladder distension), a “square” shape, a horizontal arrangement of the long axis of the gallbladder, a “whirl sign” indicating a twisted pedicle, or a “beak sign” defined as abrupt angulation of the gallbladder neck [13-16]. On hydroxy iminodiacetic acid (HIDA) scan, gallbladder volvulus may be demonstrated as a photopenic region in the right lower portion of the liver, indicating a lack of filling of the gallbladder with a focal accumulation of radioactivity medial to this area in what is known to be the cystic duct. This finding is known as a “bulls-eye” image [17]. Additionally, necrosis and hemorrhage of the gallbladder as a result of torsion may be demonstrated on magnetic resonance imaging (MRI) as high signal intensity within the gallbladder wall on T1 signal [14,18]. Lastly, magnetic resonance cholangiopancreatography (MRCP) can also be used preoperatively to diagnose gallbladder volvulus. On MRCP imaging, the extrahepatic bile duct is distorted, forms the letter “v,” and demonstrates a high-intensity signal compared to the gallbladder. The gallbladder will be deviated to the midline and observed beyond a tapered cystic duct [19].

Our patient’s imaging studies consisted of a CT scan and abdominal ultrasound completed at an outside facility. The CT noted a distended gallbladder in the pelvis with wall thickening. The radiologist indicated that a follow-up gallbladder sonogram or HIDA scan could be helpful in further evaluation. A follow-up abdominal ultrasound noted a dilated gallbladder with sludge and trace pericholecystic fluid. Although the CT scan demonstrated a low-lying gallbladder suggestive of visceroptosis, neither of the imaging studies suggested conclusive pathology specific to gallbladder volvulus. A HIDA scan and MRCP were not performed. Given our patient’s constellation of clinical, laboratory, and imaging findings, a preoperative diagnosis of acute cholecystitis was made.

To minimize the risk of gangrene and perforation, early surgical management with detorsion and cholecystectomy is the recommended treatment for patients presenting with gallbladder volvulus. Although laparoscopic cholecystectomy is widely accepted as the mainstay of treatment, robotic cholecystectomy has demonstrated promising results in the treatment of this condition and is growing in popularity [6,8,9,11-14,16,18]. With timely management of this condition, mortality is less than 5% [20]. Due to surgeon preference and the availability of the robot at our institution, a robotic cholecystectomy was performed, and excellent outcomes were achieved.

## Conclusions

In summary, gallbladder volvulus is an important condition to keep on the differential when an elderly female patient presents with right upper quadrant pain and laboratory findings suggestive of acute cholecystitis. Ultrasonography is the first-line imaging technique used to establish the diagnosis while Doppler studies should be employed when clinical suspicion for gallbladder volvulus is high. Due to the poor prognosis if managed conservatively, it is essential to employ prompt surgical intervention via laparoscopic or robotic cholecystectomy to avoid potential complications such as sepsis, gallbladder rupture, and biliary peritonitis.
